# Adaptation of Transcortical Responses in Upper Extremity Movements During an Elbow Visuomotor Tracking Task in Humans

**DOI:** 10.3390/jfmk10040368

**Published:** 2025-09-26

**Authors:** Olga Dubey, Michael A. Petrie, Richard K. Shields

**Affiliations:** Department of Physical Therapy and Rehabilitation Science, University of Iowa, 1-252 Medical Education Building, Iowa City, IA 52242, USA; olga-dubey@uiowa.edu (O.D.); michael-petrie@uiowa.edu (M.A.P.)

**Keywords:** long-latency reflex, feedback, neuromuscular control, perturbation

## Abstract

**Background**: Precise upper limb movements are essential for daily tasks and motor function. Feedforward responses enable anticipatory movement planning, while feedback responses utilize sensory information for real-time corrections. Long-latency reflexes (LLRs) represent rapid feedback responses during unexpected perturbations and are integral in maintaining motor control, yet the factors governing LLRs in the upper extremity remain unclear. **Methods**: Forty healthy participants with ages ranging from 20 to 45 years (mean = 26.75, and SD = 5.6), performed a unilateral visuomotor elbow flexion and extension task with one arm while following a sinusoidal target at varied resistances and speeds. Task performance was quantified and communicated to participants after each bout. Resistance was randomly released during the flexion phase to trigger a perturbation. Electromyography data from the biceps and triceps muscles were analyzed for the long-latency reflex (LLR) and secondarily for the short-latency reflex (SLR), and voluntary response (VR) phases. **Results**: In response to unexpected upper extremity perturbations, participants relied on two core strategies. Inhibitory LLRs within the biceps were prominent, emphasizing inhibition to maintain movement stability 50–150 ms post-disturbance. Additionally, volitional control through the triceps allowed participants to regain precision starting from over 150 ms. Participants’ responses to perturbations were dependent on speed and resistance but were not modified with learning across repeated attempts. **Conclusions**: This study reveals that participants demonstrate both long-latency and volitional responses to counteract perturbations during an upper extremity visuomotor task. These findings highlight that a predominant agonist inhibition strategy emerged during the during unpredictable perturbations of the upper extremity. Understanding these responses may inform rehabilitation and pharmaceutical interventions when treating individuals with neurological conditions that influence motor control.

## 1. Introduction

The ability to execute precise and rapid upper limb movements is fundamental to human motor function, playing a crucial role in daily tasks and high-performance actions. Human motor control is built upon the dynamic interplay of two key mechanisms: feedforward and feedback responses [1]. Feedforward responses entail pre-planned movements without real-time error correction, allowing the nervous system to predict and adjust actions to anticipated environmental changes [2]. In contrast, feedback responses rely on sensory information, such as visual, vestibular, and somatosensory inputs, to promptly detect deviations from desired trajectories and make necessary adjustments [2].

During upper extremity movements, unexpected perturbations trigger rapid feedback responses, which encompass short-latency reflexes (SLRs), long-latency reflexes (LLRs), and voluntary reactions (VRs) [3,4,5]. These responses are measured as bursts of electromyographic (EMG) activity congruent with the various latencies, depending on which extremities are perturbed. The SLR, generated approximately within the first 50 ms following a perturbation, relies on spinal networks and group I afferents [6]. In contrast, the LLR incorporates both spinal and supraspinal circuits and processes peripheral group I and group II afferents [7], emerging within 50–120 ms [8]. This response is believed to be “tuned” by transcortical feedback [9]. The voluntary reactions, initiated at approximately 120 ms post-perturbation, engage a more widespread neural network, including the premotor cortex [10] and basal ganglia [11].

LLRs, whether inhibitory or excitatory, are considered important for executing rapid and flexible corrections when faced with unexpected perturbations. Various factors influence LLR modulation, including task demands [12,13], participants’ intent [14,15], mechanical interactions across joints [16,17], limb stiffness [18], and the urgency to correct for perturbations [19]. LLRs share properties with voluntary motor control, indicating a close relationship between feedforward movement plans and voluntary reaction to a perturbation. Whether LLRs are excitatory or inhibitory may be based on environmental conditions [20] and are often more prominent in the early stages of learning [21,22,23]. Notably, LLRs appear to change with age, with older adults exhibiting larger LLRs than younger individuals [22]. With pathology, LLRs may be enhanced or attenuated based on the specificity of a lesion and the remaining feedforward control levels across a broad spectrum of neurological conditions, such as Parkinsonian syndromes [24,25], Huntington’s disease [26,27], myoclonus of varied etiologies [28,29], multiple sclerosis [30,31], and stroke [32].

Various experimental conditions and methodologies have been developed to study LLRs. These include active perturbation motors [33,34], force fields [35], and robotic devices [36,37] to impose a perturbation on an active movement. Rather than delivering a perturbation from an external torque motor or force field, we developed a method to deliver perturbations that are reminiscent of real-life conditions. For example, when the central nervous system (CNS) is fooled, like when lifting an object that is “heavier than expected” or “predicting the wrong height when stepping off of a curb”, there is an impending stretch that triggers a reflex response. In this study, we randomly released resistance during a visuomotor task at various working resistance levels, which, inherently, fooled the CNS. The LLR response to this perturbation may be delivered during stance or during upper extremity movements. Studies support that the LLR using this technology has been found to be robust and grounded by having participants’ “central set” focused on the accuracy of the visuomotor task [22,38,39,40,41].

Our previous studies using this technology during an upright weight-bearing task triggered inhibition of the agonist LLR with a robust excitation of the antagonist LLR [22], likely attributed to a fear of injury along with redundant sensory signaling from somatosensory, vestibular, and visual pathways [42,43,44]. Because the consequences of an uncorrected upper extremity perturbation may be viewed as “less urgent”, we sought to determine if an agonist inhibition strategy would supersede an antagonist excitation strategy as observed in upright postures. Importantly, few studies have focused on LLR strategies after demonstrating learning of a visuomotor task in healthy humans [21]. Accordingly, we sought to determine if upper extremity task learning occurs within a single session, whether a predominant LLR agonist inhibition (biceps) emerges for the LLR, and whether the LLR strategy changes with learning of a novel visuomotor task. We expect that the participants will show a distinct agonist inhibition (biceps) LLR and antagonist excitation (triceps) LLR, but with learning, a primarily agonist inhibition (biceps) strategy will emerge.

## 2. Materials and Methods

### 2.1. Subjects

Forty healthy participants, including 20 males, with ages ranging from 20 to 45 years (mean = 26.75, SD = 5.6), took part in this study. All participants had no ongoing orthopedic, neuromuscular, or neurological deficits or disorders. Informed consent was obtained from each participant, and the study received approval from the institution’s Human Subjects Institutional Review Board.

### 2.2. Instrumentation

**Figure 1:**
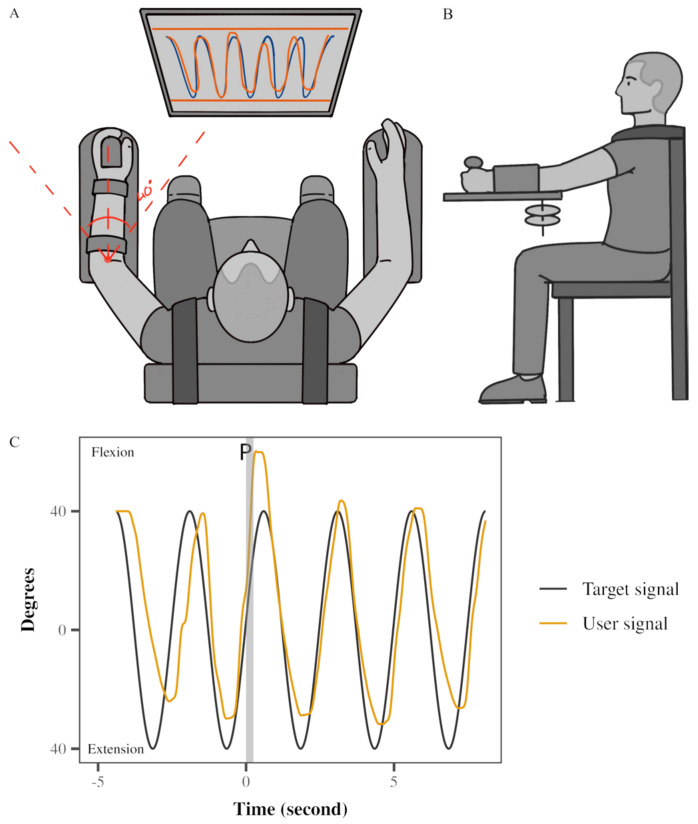
Experimental setup: (**A**) Apparatus used, the view from above; (**B**) apparatus used and experimental setup, the view from the left; (**C**) motor task: an example of real-time feedback and response to unexpected perturbation of a participant during a medium resistance and medium speed condition; black line generated by the software (target signal), yellow line generated by the participant’s left arm (user). The vertical gray bar demonstrates perturbation delivery by reducing the resistance to 0.

A Neuromuscular Therapeutic Training System (NTTS) was custom-built for the study (Figure 1A,B). The NTTS featured a gear system connected to the participants’ left upper extremity. Their arm and forearm were secured within fitted cuffs, and their hands were firmly strapped to the device. Arm flexion and extension movements caused the rack-and-pinion attachment to move horizontally. A potentiometer mounted on the gear’s shaft was calibrated for angular displacement. An electromagnetic braking system controlled the gear’s resistance, directly managed by a microcomputer through digital-to-analog input, all under customized software. The brake’s resistance was adjusted according to each participant’s left triceps MVC (maximum voluntary contraction). Minimal vertical translation of the elbow joint was possible due to the 0° to 40° elbow range of motion. Visual feedback of elbow displacement (0–40°) was displayed on a computer screen in front of the participant. The device was calibrated and showed excellent linearity, repeatability, and hysteresis of the braking and potentiometer system, all within less than 0.5% of full scale. The relationship between X (voltage) and Y (angular displacement) was determined as: y = 33.456x − 156.18. Therefore, 1 degree change would correspond to around 0.02 volts.

### 2.3. Experimental Setup

The study involved a single session using a Neuromuscular Therapeutic Training System (NTTS), delivering nine testing conditions across three attempts (sets) (see Figure 1), similar to that reported for the Single Leg Squat device [38]. These conditions combined three movement speeds (0.2, 0.4, and 0.6 Hz) with three brake resistance levels (10%, 15%, and 20% of the participant’s left triceps MVC). Testing was conducted only on the non-dominant (left) arm. A 30 s rest interval separated each testing condition. The order of testing conditions was uniform across all participants: medium, light, heavy resistance for medium speed, followed by the same resistance order at slow speed, and lastly at fast speed. Participants tracked a computer-generated sinusoidal target with five cycles. The perturbation was randomly delivered during one of the cycles between two and five (see Figure 1 with perturbation at cycle 2). Despite varying movement speeds, consistent body positions and required elbow angular control were maintained because the task was tied to a measurable human performance. Specifically, the target trajectory was always fixed across all speeds, corresponding to a range of elbow motion of 40° of elbow flexion to full elbow extension. Instantaneous visual feedback of actual elbow position (yellow sinusoidal line) was provided to subjects on the same monitor as the target trace (black sinusoidal line) (Figure 1C). No further feedback, like knowledge of results, was given other than instantaneous visual feedback and the final error score after each attempt. The subjects completed three sets of nine trials; each set, or attempt, was separated by a 5 min rest period. At the end of each set, the performance error score appeared on the computer screen and provided as feedback to motivate the participants. Participants were motivated to improve their score for each of the three attempts. A change in AE between attempts one and three was used to denote learning.

### 2.4. Performance Variables

The absolute error (AE) between the target and user traces on the visuomotor task referred to the magnitude of the discrepancy between the intended movement trajectory (represented by the target) and the actual movement trajectory performed by the user. It measured the distance between corresponding points on the target trajectory and the user’s movement position, without considering the direction of the deviation (AE = Absolute Value (Target Trace − User Trace). Peak AE provided a measure of the highest error magnitude of the AE within each flexion/extension cycle (perturbed and non-perturbed). The user rate, or slope of the user trace signal, indicated the rate of change or the speed at which the user’s movement deviated from the intended trajectory (target slope) at the time of a perturbation (user rate = (change in user trace/change in time after perturbation)∔(change in target slope/change in time after perturbation).

### 2.5. Electromyography (EMG)

We collected surface EMG signals from the muscles of the left upper extremity: (1) left triceps long head, (2) left triceps lateral head, and (3) left biceps. Before affixing the electrodes, the skin was cleaned with alcohol to ensure adequate contact. Wireless surface electrodes were placed over the muscle belly of each muscle. Once electrodes were securely placed, each subject was seated in the chair of the device with the left elbow joint positioned at 90°. The right arm was positioned in a similar position and passively supported. Subjects were then instructed to perform three maximum isometric contractions in elbow flexion and elbow extension (one at a time). Subjects were given one-minute rest between each contraction to avoid fatigue. Verbal encouragement was given during each of the contractions. The isometric MVC EMG was calculated for each muscle. We used a 100 ms RMS averaging window (2 kHz sampling rate) to smooth the signal before taking the peak. During the experiment, we smoothed all EMG using a 100 ms RMS averaging window. We next determined the peak EMG from the RMS processed signal within each bin constituting the pre-perturbation baseline (−50–0 ms), SLR (0–50 ms), LLR (50–150 ms), and VR (150–250 ms). We then normalized the peak EMG for the SLR, LLR, and VR for each condition to the pre-perturbation peak EMG for each muscle and condition. This approach enabled us to detect both excitatory and inhibitory responses relative to all experimental conditions. All EMG sampling during MVC and visuomotor tasks was performed using LabVIEW software (LabVIEW 2023 Q1, National Instruments; Austin, TX, USA).

### 2.6. Perturbation and Data Collection

Within each testing condition, the brake resistance was programmed to be removed unexpectedly for a pre-determined time equivalent to approximately 10% of the cycle duration (200, 250, and 500 ms for 0.6, 0.4, and 0.2 Hz, respectively). Brake release was randomly inserted in one of the cycles between 2 and 5, and always occurred during the early elbow flexion phase (i.e., 10° of elbow flexion) to perturb ongoing elbow flexion motion and induce stretch to the triceps. Participants completed one practice trial at medium speed before data collection.

### 2.7. Statistical Analysis

Data analysis was conducted using R Statistical Software (v4.1.2; R Core Team 2021). Prior to analysis, repeated-measures ANOVA assumptions were evaluated. Normality of residuals was assessed using the Shapiro–Wilk test, and sphericity was assessed using Mauchly’s test. When the sphericity assumption was violated, Greenhouse–Geisser corrections were applied. To examine performance measures, two-way repeated-measures ANOVAs with within-subject factors (independent variables) Time (three levels: short-latency reflex [SLR], long-latency reflex [LLR], and voluntary response [VR]) and Cycle type (two levels: perturbed vs. non-perturbed) were used. Dependent variables were peak absolute error and peak user rate. To examine left arm muscle activity, two-way repeated-measures ANOVAs with within-subject factors (independent variables) Time (SLR, LLR, VR) and Cycle type (perturbed vs. non-perturbed) were conducted on the dependent variable EMG amplitude. Learning effects were assessed separately for each time bin (pre-perturbation, SLR, LLR, VR) by utilizing two-way repeated-measures ANOVAs with within-subject factors Attempt (three levels: 1, 2, 3) and Cycle type (perturbed vs. non-perturbed) for the dependent variable peak absolute error. For the LLR time bin, two-way repeated-measures ANOVAs with within-subject factors Attempt (1, 2, 3) and Cycle type (perturbed vs. non-perturbed) were conducted for the dependent variable EMG amplitude. Significant ANOVA results lead to post hoc pairwise t-tests with Tukey’s honest significant difference adjustment for multiple comparisons. A significance level of *p* < 0.05 was used for all analyses.

## 3. Results

### 3.1. Task Performance

**Figure 2:**
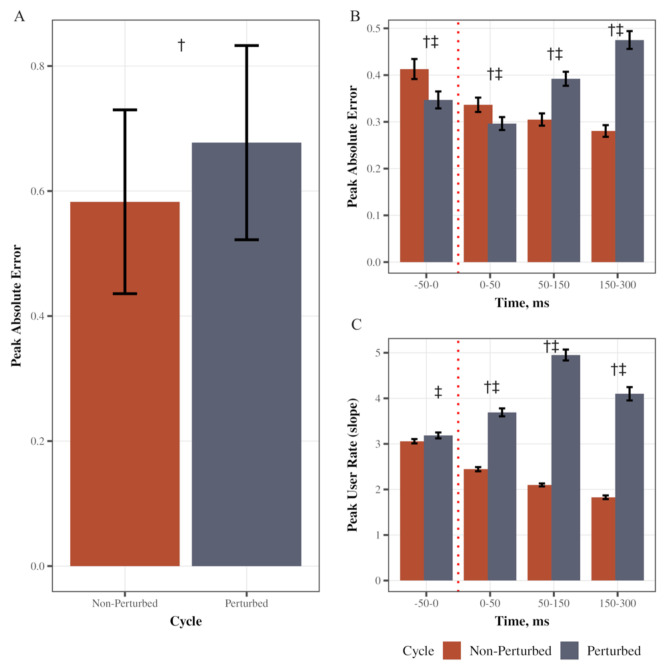
Movement performance during feedforward, feedback, and volitional reaction time bins. (**A**) Depicts the mean Peak Absolute Error across the perturbed and unperturbed cycles. (**B**) Depicts the mean Peak Absolute Error across times and during the perturbed and unperturbed cycles. (**C**) Depicts the mean Peak User Rate across times and during the perturbed and unperturbed cycles. Error bars are standard error (SE). † Depicts that the error or user rate was significantly different in the perturbed cycle compared to the non-perturbed cycle. ‡ Depicts that the error or user rate was significantly different from all other time bins within the perturbed cycle.

We first verified that the perturbation increased error in performance during the visuomotor task. Peak absolute error was higher in perturbed cycles compared to non-perturbed (main effect of cycle type: F(1, 39) = 80.11, *p* < 0.0001, partial η^2^ = 0.67, Figure 2A). Specifically, there was a significant Cycle type (perturbed/non-perturbed) × Time (pre, SLR, LLR, VR) interaction (F(1.33, 51.90) = 159.67, *p* < 0.0001, partial η^2^ = 0.80; Greenhouse–Geisser corrected; Figure 2B). Post hoc pairwise comparisons with Tukey’s adjustment indicated that in perturbed cycles, peak absolute error was higher in the LLR and VR time bins as compared to other bins (LLR vs. SLR, LLR vs. VR, and SLR vs. VR *p* < 0.0001; LLR-pre-perturbed *p* = 0.0006, SLR-pre-perturbed *p* = 0.0001), supporting that the perturbation increased error in performance. There also was a significant Cycle type × Time interaction (F(1.96, 76.38) = 262.52, *p* < 0.0001, partial η^2^ = 0.87; Greenhouse–Geisser corrected; Figure 2C). Post hoc pairwise comparisons with Tukey’s adjustment indicated that the peak user rate (user slope) was higher in all time bins after a perturbation during perturbed cycles (SLR, LLR, and VR; *p* < 0.0001) as compared to cycles without perturbation. In the perturbed cycles, peak user rate increased after the perturbation (SLR vs. pre-perturbed, LLR vs. SLR *p* < 0.0001) and then decreased in VR time (VR vs. LLR *p* < 0.0001). The magnitude of the increase in error during the unexpected perturbation assured us that the mechanical release of the braking device induced a robust perturbation allowing us to study the impact on the triggered EMG responses.

### 3.2. Synergistic Strategy in Response to the Unexpected Perturbation

**Figure 3:**
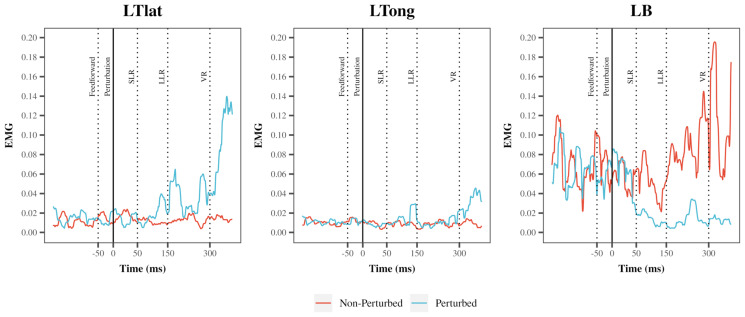
An example of a root mean square (RMS) transformed EMG signal during a perturbed and non-perturbed cycle in a single participant. A representative example of an RMS transformed EMG signal of left triceps lateral head (LTlat), left triceps long head (LTlong), left biceps (LBs) during condition 6 (medium speed + medium resistance). Outline of time periods used for EMG analysis: Feedforward: −50 ms to −1 ms prior to perturbation; Short-Latency Reflex (SLR): 1–50 ms following perturbation; Long-Latency Reflex (LLR): 50–150 ms following perturbation, Voluntary Reaction (VR): 150–300 ms following perturbation.

An example of a single participant’s EMG in the left lateral head of the triceps, long head of the triceps, and the biceps is depicted in Figure 3.

**Figure 4:**
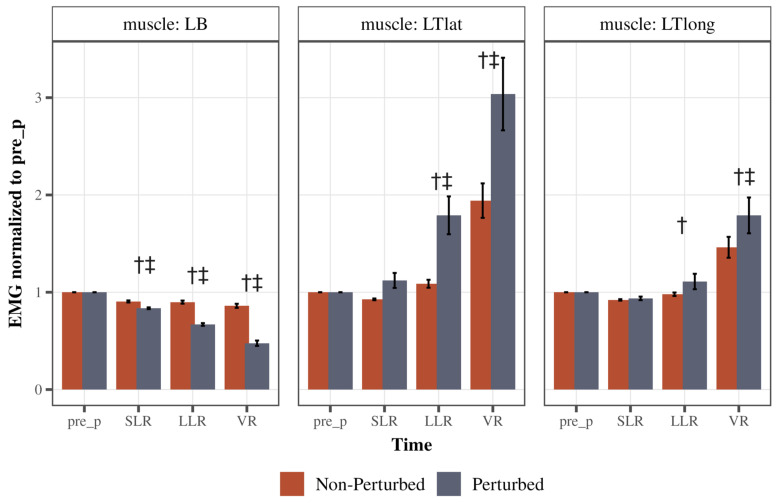
Comparison of response strategies of the left arm. The left graph depicts left biceps (LBs) peak EMG activity across time during the perturbed and unperturbed conditions. The middle graph depicts the left triceps lateral head (LTlat) peak EMG activity across time during the perturbed and unperturbed conditions. The right graph depicts left triceps long head (LTlong) peak EMG activity across time during the perturbed and unperturbed conditions. EMG activity was normalized to the pre-perturbed activity for each respective muscle. Error bars are standard error (SE). † Depicts that muscle activity was significantly different in the perturbed cycle compared to the non-perturbed cycle. ‡ Depicts that muscle activity was significantly different from the pre-perturbed activity within the perturbed cycle.

There was a significant Cycle type (perturbed/non-perturbed) × Time (pre, SLR, LLR, and VR) interaction for both triceps heads: the lateral head (LTlat: F(1.31, 51) = 18.69, *p* < 0.0001, partial η^2^ = 0.32, Greenhouse–Geisser corrected) and the long head (LTlong: F(1.27, 49.53) = 8.03, *p* < 0.0001, partial η^2^ = 0.17, Greenhouse–Geisser corrected; Figure 4 middle and right panels, respectively). Specifically, the LLR and VR for the lateral head of the triceps were significantly increased in perturbed cycles as compared to the non-perturbed (both *p* < 0.0001, Figure 4 middle panel). Within the perturbed cycles, lateral head of the triceps activity was higher during LLR and VR time compared to pre-perturbed time (*p* = 0.0003 for LLR, *p* < 0.0001 for VR). For the long head of triceps, similarly, LLR and VR activity were higher during the perturbed cycles as compared to non-perturbed (*p* = 0.049, *p* < 0.0001, respectively, Figure 4 right panel). Within the perturbed cycles, long head of the triceps activity was higher during VR time compared to pre-perturbed time (*p* < 0.0001), but not LLR (*p* = 0.69).

There was a significant Cycle type (perturbed/non-perturbed) × Time (pre, SLR, LLR, and VR) interaction for the left biceps (LBs: F(1.39, 54.25) = 273.35, *p* < 0.0001, partial η^2^ = 0.88, Greenhouse–Geisser corrected; Figure 4 left panel). Left biceps activity was lower during SLR, LLR, and VR time bins of perturbed cycles compared to non-perturbed (all *p* < 0.0001). Within the perturbed cycles, the left biceps’ EMG activity gradually decreased after the perturbation compared to pre-perturbation activity, demonstrating an inhibitory LLR and VR (both *p* < 0.0001). These findings indicate that there was a combined inhibition of the biceps, or the agonist, associated with an excitation to the antagonists, or the lateral head and long head of the triceps, during the LLR and the VR time.

**Figure 5:**
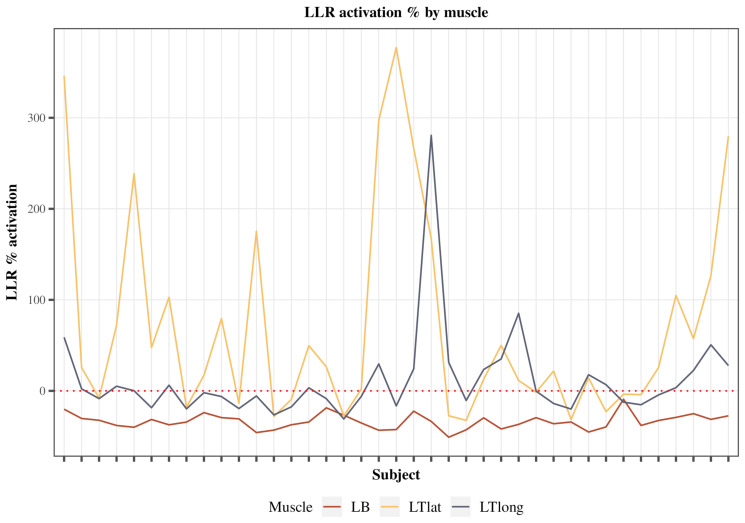
Electromyography percent change in the LLR activity from the pre-perturbed activity of the Agonists and Antagonists for all subjects. Percent change was calculated as follows: activation percent = perturbed LLR EMG − pre-perturbed EMG/pre-perturbed EMG × 100% for each respective muscle. Three of the following muscles were measured, including left biceps (red), left triceps lateral head (yellow), and left triceps long head (dark).

The investigation focused on the percentage change in LLR activity from the pre-perturbed baseline for both the agonist and antagonist muscle groups (Figure 5). Notably, the biceps muscle exhibited a characteristic trend of inhibition across all subjects, as indicated by a negative percent change, denoting a reduction in LLR activity relative to its pre-perturbed baseline. The mean left biceps inhibition across all subjects was 33.58% (SE = 0.89). Conversely, both triceps muscles displayed activation, evident through positive percent increase values, signifying an enhancement of LLR activity in response to the perturbation. The mean left triceps lateral head activation across all subjects was 79.07% (SE = 14.28). The mean left triceps long head activation across all subjects was 11.03% (SE = 4.93). Notably, the biceps inhibition strategy was less variant than the triceps excitation strategy.

### 3.3. Learning Effect and Strategy Refinement

**Figure 6:**
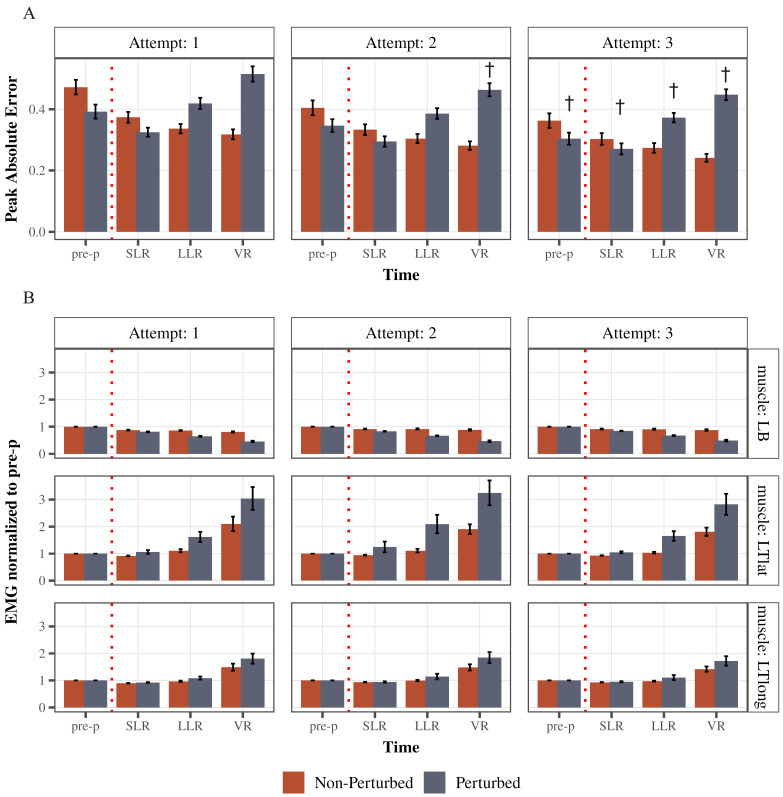
Learning effect over trial sessions (attempts) one, two, and three. (**A**) Illustrates the mean peak Absolute error across time and across attempts 1, 2, and 3 (left panel, middle panel, and right panel, respectively) for perturbed and non-perturbed cycles. (**B**) Illustrates the average response strategies employed by all subjects across attempts 1, 2, and 3 (left panel, middle panel, and right panel, respectively) for perturbed and non-perturbed cycles. Each bar represents the mean peak EMG for each time bin. EMG activity was normalized to the pre-perturbed activity for each respective muscle. Error bars are standard error (SE). † Depicts that the peak Absolute error was significantly different from attempt 1.

The testing paradigm provided illustrates rapid learning through three attempts (Figure 6A). As performance was improving, the mean peak absolute error was decreasing both in non-perturbed cycles (main effect of attempt: F(1.78, 69.56) = 17.84, *p* < 0.0001, partial η^2^ = 0.31, Greenhouse–Geisser corrected) and in perturbed cycles (main effect of attempt: F(1.95, 75.91) = 11.79, *p* < 0.0001, partial η^2^ = 0.23, Greenhouse–Geisser corrected). Specifically, post hoc comparisons indicated that in perturbed cycles, peak absolute error in pre-perturbation time bin decreased from attempt 1 to attempt 3 (*p* = 0.0008), as well as in SLR time bin (attempt 1 to 3 *p* = 0.02), LLR time bin (attempt 1 to 3 *p* = 0.04) and VR time bin (attempt 1 to 2 *p* = 0.03; attempt 1 to 3 *p* = 0.003).

As for the muscle activity adaptations (Figure 6B), a single testing session resulted in modulation of left triceps lateral head (LTlat) activity in the LLR time bin after a perturbation: it increased from 1.62 ± 0.18 (SE) to 2.09 ± 0.34 during the second attempt and then decreased to 1.65 ± 0.18 in the third attempt. However, this difference was not statistically significant across attempts (F(1.24, 48.42) = 2.02, *p* = 0.16, partial η^2^ = 0.05, Greenhouse–Geisser corrected). As for the long head of triceps, it followed a similar pattern (1.08 ± 0.05 during first attempt; 1.14 ± 0.1 during second attempt; 1.10 ± 0.09 during third attempt)—not significant across attempts (F(1.23, 47.9) = 0.47, *p* = 0.54, partial η^2^ = 0.12, Greenhouse–Geisser corrected). Left biceps LLR activity was increasing slightly through attempts (0.65 ± 0.02 first attempt; 0.67 ± 0.01 second attempt; 0.68 ± 0.01 third attempt) but not significant across attempts (F(1.68, 65.59) = 2.5, *p* = 0.09, partial η^2^ = 0.06, Greenhouse–Geisser corrected).

## 4. Discussion

LLR inhibition of the agonist was the predominant strategy utilized to stop upper extremity flexion after an unexpected perturbation in this study. The LLR inhibition of the bicep agonist was invariant, as 100% of all participants demonstrated this inhibition (Figure 5). LLR excitation of the long and lateral head of the triceps, while statistically significant, was more variable and elicited by 53 and 33 percent of all participants, respectively. These findings are in contrast to the predominant antagonist excitation strategy observed in our lower extremity weight-bearing studies [22,38,44]. This predominant inhibition strategy of the biceps and excitation of the triceps did not change after the participants significantly improved their task performance (Figure 6). Taken together, these findings suggest that differences in task urgency may explain LLR strategies in the upper and lower extremities [19,21,22,44,45] and improved performance, even to perturbations, does not necessarily require feedback control modulation. Future studies are needed to evaluate the relative influence of feedforward control strategies on feedback control and their association with rapid upper extremity learning.

### 4.1. Feedforward and Feedback Movement Control Strategies

The human ability to track moving objects is essential for everyday activities, such as driving, catching a ball, and using a computer mouse. Visuomotor tracking is the process of using visual information to control the movement of an arm or hand to track a moving target. The brain uses a combination of feedback and feedforward control to achieve accurate tracking. Feedback control involves using visual information about the error between the current position of the hand and the target position to adjust the motor commands. Feedforward control involves using stored information about the target motion to generate motor commands before the target moves. The motor control system must be able to resist perturbations in a wide range of external conditions, ranging from isometric to inertial or compliant loading. This can include situations of position maintenance as well as impending or ongoing movement. The challenge is multiplied when several degrees of freedom are involved, and many directions of perturbation are considered [46]. Examples of perturbations might include sudden pushes or pulls, changes in surface or terrain, or unexpected obstacles in the environment [47]. The methods employed in our study simulate real-world scenarios of injury by incorporating a perturbation paradigm that mimics sudden changes in resistance during active movement [44]. Such abrupt perturbations resemble situations where unexpected forces, such as slips, sudden pulls, or loss of support, can occur in daily life or athletic activities. By utilizing the NTTS in this study, we introduced unanticipated brake releases during elbow flexion, and we could replicate the mechanical conditions that are associated with muscle strain or joint injury [42,43].

Recent computational theories of motor coordination suggest that control signals are derived online based on task-specific cost functions [5,48]. This theory is based on stochastic optimal feedback control, which provides a computational framework for understanding how the nervous system might account for variations in environmental and internal conditions. The nervous system derives optimal control signals not only to respond to changes in task goals itself, but it also tunes limb impedance to ensure that any perturbations arising after task goals have been countered in a task-specific manner [49]. The tuning of reflex circuits in accord with changing task goals is one mechanism to confer such extraordinary specificity. The minimum intervention principle [50] within the optimal feedback control framework suggests that the controller intervenes to counter perturbations that interfere with task goals but does not resist perturbations that assist goal achievement. This may explain the predominant use of volitional response in studies, as participants may not perceive the perturbation to the upper extremity as an “urgency risk” requiring immediate rectification. The LLR agonist inhibition strategy that we observed in this study was in contrast to the more robust LLR antagonist excitation strategy evoked during single limb stance perturbations that were congruent with a higher “urgency risk” related to injury [44]. Because we used an identical method (NTTS) in both studies, we provide a unique contrast across two important functional tasks that have distinctly unique sensory triggering mechanisms.

### 4.2. Theoretical Mechanisms Modulating LLRs

It is well known that background activity of alpha motor neurons in the target muscle or co-contraction of antagonist muscles [51,52] leads to increased stiffness and may trigger larger reflex responses, while a decrease in background muscle activity results in smaller reflex responses, indicating a direct relationship between response amplitude and baseline muscle activation.

Another strategy to gain these pathways includes changes in the activation of gamma motor neurons [53,54,55,56,57,58,59,60]. Gamma motor neurons, activated alongside alpha motor neurons, sustain the firing of spindle afferents when the extrafusal muscles contract [61] and are adjusted during difficult tasks [62]. Dynamic gamma activity may modulate Ia afferent and stretch reflex sensitivity if the movement direction stretches the spindle-bearing muscle, allowing for independent tuning of muscle stiffness [63]. Human studies have supported the co-activation of alpha and gamma motor neurons and the velocity-signaling role of Ia afferents, while also suggesting simultaneous inhibition of antagonist gamma motor neurons according to the demands of the motor task [64,65]. Importantly, due to the transcortical nature of the LLR, the Ia, II, and Ib inputs may be modulated in a supraspinal manner via a pathway separate from α commands to muscles, with recent data suggesting that this alternative pathway involves γ commands to muscle spindles [66]. While we were not able to align our findings to this level of mechanistic specificity, we are reminded of the many opportunities for supraspinal modulation of peripheral responses to perturbations.

Other intriguing theoretical mechanisms involve pre-synaptic action of descending neurons onto incoming afferents or inter-neuronal networks that may modify reflex pathway output. Depolarization of afferents projecting to the spinal cord inhibits neurotransmitter release, effectively diminishing or preventing activation of motor neurons innervated by these afferents [67,68,69,70,71]. More recent research by Seki et al. [72] demonstrated such a mechanism for modulating cutaneous reflexes. Importantly, LLRs are also influenced by the mechanical stability of the environment. The nervous system adjusts LLRs to compensate for environmental instabilities, such as changes in arm orientation or increased muscle co-activation to increase stiffness [73,74]. LLRs can be scaled up or down based on the stability of the environment, providing additional restoring forces to reinforce stable behavior [75,76]. Based on these mechanisms, we appreciate that more precise quantification of synergy stiffness, as a function of learning, may offer rich future opportunities to modulate alpha, gamma, and pre-synaptic inhibitory pathways in human studies.

### 4.3. Learning and Reflex Responses

During the process of skill acquisition, there is a shift in movement control from relying on feedback to relying on feedforward mechanisms. With practice, movements become swifter and more precise, and the learner gradually reduces their dependence on feedback control [77]. At the outset, participants exhibited a range of responses in managing the unexpected perturbations induced by the triceps stretch in this study. These strategies often reflected an initial understanding of the task and expectations, as participants grappled with the unfamiliar nature of the perturbations. However, as the experiment progressed, distinct patterns of refinement emerged, indicating an ongoing learning process. As participants gained experience through repeated attempts, there was a robust shift in response performance. While initial responses were sometimes less coordinated and varied, subsequent trials revealed a consistent trend towards more precise and efficient strategies, as supported by the progressive improvement in performance error.

The magnitude of the stretch required to produce a long-latency reflex in the triceps muscle can vary depending on factors such as the individual’s age, muscle condition, and the characteristics of the stretch. However, in general, a stretch of approximately 2–4% of the muscle’s resting length is thought to be sufficient to evoke a long-latency reflex in the triceps muscle [78]. For example, a study by Bedingham and Tatton [79] found that a stretch of 15% of the resting length was required to produce a long-latency reflex in the triceps muscle of healthy adults. It has been shown that the size of the stretch response is a direct function of the log velocity of the stretch [25]. However, one study found that the magnitude of the triceps’ spinal stretch reflex was modulated based on the magnitude of elbow extension needed to return the hand to the target, rather than the amount the triceps was stretched [80], which may explain the consistency of the agonist inhibition strategy over the antagonist excitation strategy observed in this study. Another study [81] discovered that at 0° shoulder elevation, the long head of the triceps brachii generates a significantly higher muscle force and muscle activation than the lateral and medial heads. Meanwhile, at 90°, 135°, and 180° shoulder elevation, the medial head of the triceps brachii showed a significantly higher muscle force than the long and the lateral heads. Considering this mechanical advantage, the long and lateral heads of the triceps might not have been optimal to trigger a robust stretch.

In the context of a voluntary movement involving a single joint, it has been observed that the stretch reflex response is weakened, with a gradual recovery occurring later in the movement [82]. Even during slower movements, the inhibition of the myotatic reflex is still observed. The extent of suppression is related to the speed of the movement, and the duration of this suppression consistently extends beyond the duration of the movement itself. This attenuation of the reflex response is accompanied by a decrease in joint stiffness [83], indicating that the neural response is not optimally prepared to counteract external disturbances or perturbations.

When the external force field has the potential to destabilize the arm by amplifying any deviation of the hand from the intended movement direction, the participant adopts a proactive strategy to accomplish the task. Instead of altering the joint torques, they increase the co-contraction of antagonist muscles to enhance stiffness in the direction of instability [84,85]. Through the attempts, subjects were adjusting their response strategy: increasing LLRs and volitional response at first, and subsequently shifting to a more feedforward strategy, potentially using co-contraction and maintaining a certain level of muscle tone.

Other considerations, such as the perceived risk and context of the task, seemed to have a more dominant influence on the chosen response strategies. If we define stability as the ability to prevent dangerous situations such as falls, collisions, or injuries when responding to external forces, then the motor control system should prioritize avoiding such events. In normal circumstances, the system has the capability to achieve this goal, and it typically succeeds [47]. The seated nature of our study, as compared to previous investigations involving weight-bearing tasks, may contribute to the differences in findings. This distinction underscores the significance of the integration of vestibular, somatosensory, and visual feedback in determining the extent of the long-latency reflex during seated conditions. Therefore, the mechanism of counteracting unexpected perturbation in movement in the upper limb appears different compared to the lower limb [38]. Our observations point to the contextual nature of the task, where the perceived urgency did not warrant the utilization of exclusively a long-latency response strategy. The potential for harm if the arm was not stopped immediately was not seen as significant. Therefore, participants opted for a deliberate and controlled response, reflecting their perception that there was no imminent threat requiring rapid action. Moreover, the distinct role of the lateral triceps muscle in response strategies was evident. While contributing to volitional responses, it did significantly contribute to reflexive responses to the induced perturbation. Interestingly, the dominance of LLR inhibition of the biceps as one of the primary strategy choices for this task emerges as a notable pattern. This preference for inhibiting the agonist muscle aligns well with the antigravity nature of the task, where inhibition emerges as a favorable mechanism for successful response execution.

The observed learning effect and strategy refinement hold implications for our understanding of motor learning and adaptation. The refined responses suggest that participants not only adjusted to the task’s demands but also leveraged their experience to optimize their strategies. The evolving nature of these responses underscores the cognitive dimension of motor control, where participants navigated between volitional and reflexive strategies in a context-dependent manner.

## 5. Limitations

We were unable to measure specific alpha, gamma, pre-synaptic, post-synaptic, or supraspinal mechanisms that may underly the findings of this study. We discuss these areas from a speculative perspective rather than from a causal perspective. We also chose to keep the order of the nine conditions constant across the three attempts offered in our study. Although our perturbations were random within each condition, the order of the conditions (speed and resistance) may have contributed to the learning process across the three attempts. We had a balanced design in this study with an equal number of males and females, but due to the extensive analysis in this report, we elected to focus on the overall generalizability of the findings and not include biological sex. Future analyses are underway to examine the impact of biological sex on transcortical responses during visuomotor tasks. Lastly, the insignificant change in reflexes after learning may have been present, but small, and not detectable given the power of this study. Future studies with larger samples are needed to build on these findings to advance our understanding of reflex modulation after motor learning.

## 6. Conclusions

In this study, we discovered that an LLR agonist inhibition coupled with an antagonist excitation was triggered by perturbations of the upper extremity, and the strategy did not change after learning a novel visuomotor task. Overall, inhibition of the agonist (biceps) was the strategy used by all participants in conjunction with less frequent antagonist excitation. Learning a novel task in this study may have been driven by a modified feedforward control strategy rather than relying on a feedback control strategy, but this finding will require further confirmation in future studies. These findings provide a framework to further assess new rehabilitation interventions and/or pharmacological therapies to understand movement control challenges for people with neurological disorders.

## Data Availability

Data will be made available upon reasonable request.

## Abbreviations

The following abbreviations are used in this manuscript:

SLR	Short-latency response
LLR	Long-latency response
VR	Volitional response
EMG	Electromyography
